# p120-catenin phosphorylation status alters E-cadherin mediated cell adhesion and ability of tumor cells to metastasize

**DOI:** 10.1371/journal.pone.0235337

**Published:** 2020-06-26

**Authors:** Alisha M. Mendonsa, Chirosree Bandyopadhyay, Barry M. Gumbiner

**Affiliations:** 1 Center for Developmental Biology and Regenerative Medicine, Seattle Children’s Research Institute, Seattle, Washington, United States of America; 2 Department of Biochemistry, University of Washington, Seattle, Washington, United States of America; 3 Department of Pediatrics, University of Washington, Seattle, Washington, United States of America; University of Alabama at Birmingham, UNITED STATES

## Abstract

p120-catenin is considered to be a tumor suppressor because it stabilizes E-cadherin levels at the cell surface. p120-catenin phosphorylation is increased in several types of cancer, but the role of phosphorylation in cancer is unknown. The phosphorylation state of p120-catenin is important in controlling E-cadherin homophilic binding strength which maintains epithelial junctions. Because decreased cell-cell adhesion is associated with increased cancer metastasis we hypothesize that p120-catenin phosphorylation at specific Serine and Threonine residues alters the E-cadherin binding strength between tumor cells and thereby affect the ability of tumor cells to leave the primary tumor and metastasize to distant sites. In this study we show that expression of the p120-catenin phosphorylation dead mutant, by converting six Serine and Threonine sites to Alanine, leads to enhanced E-cadherin adhesive binding strength in tumor cells. We observed a decrease in the ability of tumor cells expressing the p120-catenin phosphorylation mutant to migrate and invade using *in-vitro* models of cancer progression. Further, tumor cells expressing the phosphorylation mutant form of p120-catenin demonstrated a decrease in ability to metastasize to the lungs using an *in-vivo* orthotopic mammary fat pad injection model of breast cancer development and metastasis. This suggests that regulation of p120-catenin phosphorylation at the cell surface is important in mediating cell-adhesion, thereby impacting cancer progression and metastasis.

## Introduction

E-cadherin, the epithelial adhesion molecule, has been known to play an important role in cancer development and metastasis. A decrease or loss of E-cadherin expression in tumor cells has been linked to epithelial to mesenchymal transition (EMT) where cells lose their epithelial phenotype and gain a more migratory mesenchymal phenotype. The loss of cell junctions and cell adhesion mediated by the loss of E-cadherin binding between cells enables tumor cells to dissociate from the primary tumor, invade surrounding tissues and migrate to distant sites and establish metastatic tumors. E-cadherin expression can also be altered through accumulation of mutations, loss of heterozygosity and epigenetic regulation of its expression resulting in promoter methylation. Loss of E-cadherin expression can promote tumor cell invasion and metastasis whereas increased expression of E-cadherin has been shown to reverse these phenotypes [1–5].

While EMT and decreased E-cadherin levels can explain some cases of cancer progression, there are still instances where cancer cells maintain E-cadherin expression on their cell surface, do not undergo EMT and are still able to facilitate metastatic outgrowth at a distant site [6–8]. It has been shown that E-cadherin expression is maintained in circulating tumor cell clusters and this enhances tumor cell survival and collective migration of tumor cells [7]. E-cadherin missense mutations are observed in patients with hereditary diffuse gastric cancer and these mutations are thought to be causative for cancer development [9]. While most of these mutations result in truncations and loss of E-cadherin mediated cell adhesion, there are still some missense mutations that are expressed on the cell surface and retain cell adhesive function [10]. Therefore, while there is evidence that E-cadherin is still expressed in several types of cancers, it is not fully understood how E-cadherin mediated cell adhesion is regulated and altered as cancer progresses and metastasizes.

E-cadherin is bound to α-catenin, β-catenin, and p120-catenin through its cytoplasmic tail. This cadherin-catenin complex creates a bridge between E-cadherin and the actin cytoskeleton and can mediate both inside-out and outside-in signaling between cells [11, 12]. The binding of p120-catenin to the E-cadherin juxta membrane domain is known to regulate E-cadherin surface levels and control E-cadherin protein turnover by suppressing endocytosis [13, 14]. p120-catenin is a member of the armadillo-repeat family of proteins and has N-terminal coiled-coil and regulatory domains [15]. Within the p120-catenin regulatory domain lies a phosphorylation domain that harbors eleven serine, threonine and tyrosine phosphorylation sites [16, 17]. Src family kinases, PKC and EGFR have been shown to be important in mediating changes in p120-catenin phosphorylation [18]. Although the phosphorylation state of p120-catenin does not generally influence E-cadherin stability, it can regulate the strength of the E-cadherin homophilic bond and thus regulate E-cadherin mediated cell adhesion and adhesive strength [11, 12]. When p120-catenin is phosphorylated, E-cadherin is in a low adhesion state while dephosphorylation of p120-catenin leads to strong E-cadherin adhesive binding, providing one mechanism for controlling the level of adhesion between cells [19].

p120-catenin has been considered a tumor suppressor as a result of its ability to stabilize E-cadherin at the cell surface. Several studies have shown that p120-catenin mis-localization or loss indeed results in pro-tumorigenic events [20–22]. In an APC min model, it was shown that p120-catenin is an obligate haploinsufficient tumor suppressor in intestinal neoplasia indicating that p120-catenin expression levels can control tumorigenicity [21]. Recent studies have also shown that signaling events downstream of p120-catenin and cadherins are crucial for tumorigenicity including Src-mediated transformation as a result of p120-catenin phosphorylation [16, 23]. Although *in-vitro* evidence suggests a pro-tumorigenic role for p120-catenin phosphorylation, the mechanism underlying this role is largely unknown. The p120-catenin Y228 phosphorylation has been correlated with progression of oral squamous cancer and aggressiveness of glioblastoma [18, 24, 25]. Tyrosine and threonine phosphorylation of p120-catenin in two sites, Y228 and T916, have been observed to be elevated in renal and breast tumor tissue samples [18]. However, a detailed understanding of what p120-catenin does, how its phosphorylation is controlled and what are the implications in cancer progression have not been evaluated.

We have shown that multiple Serine/Threonine residues are dephosphorylated when E-cadherin adhesion is enhanced either by use of E-cadherin activating antibodies or inside-out activation through Nocodazole or LiCl in colo205 cells [11, 12]. Multiple phosphorylation residues were tested either alone or in combination and it was found that the 6 Serine/Threonine residues (S252, S268, S288, T310, S312, and T916) in the p120-catenin regulatory domain were important in this process. We testing the effects of mutating these six residues to alanine on cell adhesion and cell migration in both colorectal and epidermal tumor cell lines, and found that expression of the non-phosphorylatble mutant increased E-cadherin adhesive strength and reduced cell migration [12]. The aim of the present study is to evaluate the role of p120-catenin phosphorylation state on E-cadherin mediated cell adhesion in cancer development and progression to metastasis. We thus hypothesized that dephosphorylation of p120-catenin leads to enhanced E-cadherin mediated cell-adhesion and a decrease in the ability of tumor cells to metastasize. Here we show that expression of the p120-catenin Serine/Threonine phosphorylation dead mutant (S/T6A) leads to increased E-cadherin mediated cell adhesion when expressed in the 4T1 breast cancer cells. The 4T1 cell line was used as it has been previously demonstrated that these cells undergo tumor metastases while retaining E-cadherin cell surface expression, which would be necessary to test our hypothesis [26, 27]. Expression of the p120-catenin phosphorylation dead mutant led to decreased ability of tumor cells to migrate and invade *in-vitro*. Further, tumor cells expressing the p120-catenin S/T6A phosphorylation dead mutant had fewer metastases to the lung when injected using an orthotopic mammary fat pad injection compared to tumor cells expressing Wildtype (WT) p120-catenin isoform 3. Thus, changes in p120-catenin phosphorylation can alter the adhesive state of E-cadherin at the cell surface and decrease the metastatic transformation of tumor cells.

## Materials and methods

### Establishment of stable 4T1-Luc2 p120-catenin phosphorylation mutant cell lines

4T1-Luc2 cells were obtained from the ATCC(CRL-2539-LUC) and maintained in RPMI Medium 1640 supplemented with 10% FBS. Cell lines were checked every 6 months for mycoplasma contamination. To knockdown endogenous p120-catenin, shRNA from the Broad Institute library targeting the 3’UTR for p120-catenin was obtained (GCTGCCGTCATCCGTGAATTA) in the pLKO_TRC005 lentiviral vector. Lentiviral particles and control particles were produced in HEK293T17 cells and used to transfect 4T1-Luc2 cells. Post-transfection, shRNA-expressing cells were cloned by serial dilution. Knockdown was confirmed in resulting clones with western blot analysis. Using the Gateway cloning system, either WTp120-catenin isoform 3, or the S/T6A p120-catenin isoform 3 mutant that includes S252A, S268A, S288A, T310A, S312A, and T916A mutations were introduced into plx304 lentiviral vector with Blasticidin resistance. Lentiviral particles were produced in HEK293T17 cells and used to transfect one p120shRNA cloned 4T1 cell line (shP120) and selected with Blasticidin. Single cell clones were FACS sorted based on E-cadherin surface expression and used for subsequent experiments.

### Western blot

Protein lysates were prepared with Digitonin lysis buffer. Protein concentration was determined by the Pierce BCA protein assay (Thermo Scientific). 30 μg of protein was loaded into each well and separated on a 5–17% SDS-PAGE gel. Proteins were transferred onto a PVDF membrane, subsequently blocked with 3% milk, and then incubated with anti E-cadherin at 1μg/ml (Produced in house [28]) or anti p120-catenin antibody at 1:2000 dilution (BD 610133) overnight at 4°C. The blots were washed and then incubated with secondary antibody (IR700 conjugated goat anti rabbit IgG (LIC-926-68021)/ IR800 conjugated goat anti mouse IgG (LIC-926-32210) at 1:10,000 dilution) for 1 h at room temperature. Blots were washed and then imaged with Licor Odyssey scanner. For phosphorylation analysis, cell were treated with E-cadherin functional antibodies for 24 hours at 3μg/ml. Protein lysates were resolved by 6% SDS-PAGE using 37:5:1 bis-acrylamide with 20μM Phos-tag^®^ acrylamide and 10 mM MnCl_2_ running at 80 V. Blots were imaged with a bio-rad chemidoc.

### Flow adhesion assay

The flow cell adhesion assay was conducted as described previously [29]. Briefly, the cells were dissociated in the presence of 0.2mM EDTA. Cells were washed and resuspended in Hank’s Balanced Salt Solution (HBSS)(Gibco-14025092). Cells were then allowed to attach to glass capillary tubes coated with human E-cadherin extracellular domain for 10 minutes. Using an effusion pump, cells were washed away with HBSS at the indicated increasing flow rates for 30 second increments. The number of cells remaining attached to the capillary after each increment was counted, and the percentage of cells that remained adhered to the capillary was calculated.

### Cell viability assay

Viability of tumor cells was determined using the CellTiter aqueous non-radioactive cell proliferation assay (Promega G5421) according to the manufacturer’s protocol. Briefly, 10×10^4^ cells were plated in each well of a 96 well plate and allowed to attach overnight. Cells were serum starved for 24 hours followed by changing to DMEM containing 10% FBS for 24 hours. 20 μl of MTS reagent was added to each well and incubated at 37°C for 4 hours. Absorbance at 490 nm was read using a SpectraMax i3x microplate reader. Experiments were carried out in triplicate with 3 replicates per plate.

### BrdU incorporation assay

Proliferation of cells was measured with the BrdU proliferation kit (Millipore-Sigma 2750) and was carried out as per the manufacturers protocol. This non-radioactive system detects the amount of BrdU within chromosomal DNA by peroxidase-labeled anti-BrdU antibody-colored 3,3',5,5'-tetramethylbenzidine. Cultured cells without BrdU addition were used as controls for non-specific binding. Optic absorption at 450 nm was measured to detect BrdU, using a plate reader (SpectraMax i3x microplate reader). Three independent experiments were performed, and the extent of proliferation was expressed as OD mean values in the presence of BrdU ± SEM of triplicate wells.

### Transwell migration and invasion assays

Cell migration and invasion were assessed by a modified Boyden assay using 24 well multi-well inserts (Millipore) with 8 μm pore PET membrane. Cells (1×10^5^ cells/chamber) were resuspended in media plus 1% FBS directly on the insert membrane (Migration) or in 100 μl of 1 mg/ml of basement membrane matrix (BD) (Invasion) and seeded on the membrane. 10% FBS supplemented media was added to the lower chamber and cells were allowed to migrate through the filter for 16 h at 37°C in 5% CO_2_. Cells on the top of the membrane were scraped off with a Q-tip. Migrated or invaded cells on the lower surface of the membrane were fixed in 100% methanol, stained with Hoechst, and imaged. Cells that fell to the bottom of the 24 well plate were fixed in 100% methanol, stained with Hoechst, and imaged. Experiments were carried out in triplicate with three replicates per experiment and the number of cells migrated/invaded per 10X field was determined from five random fields per well.

### 3D invasion assay

The 3D invasion assay was carried out as described by [30]. Spheroids are generated in hanging drop cultures for 72 hours by pipetting 20 μl drops with 500–1000 cells per drop on the tissue culture dish lid. The lid is upturned onto the plate with 5ml of PBS to prevent the hanging drops from drying out. Spheroids are collected by tilting the dish and pooling the media and transferring to a 1.5 ml microcentrifuge tube and allowing the spheroids to settle. To embed the spheroids, a 1:1 mixture of Matrigel and type I collagen was used and combined with the spheroids in a 1:5 ratio and 40 μl added onto each well of a 24-well plate. After 30 min, media was added to the wells. Plates were incubated at 37°C and 5% CO_2_ incubator for 72 hours and then imaged. Distance invaded was measured from the circumference of the sphere to the end of the invasive front. The longest distance invaded per sphere and average of all invasive protrusions (multiple invasive protrusions per sphere) of 10 randomly chosen spheroids per cell type were measured. The experiment was repeated 3 times.

### Animal care

All animal procedures were conducted in accordance with Guidelines for the Care and Use of Laboratory Animals. Experimental procedures and protocols were approved by the Seattle Children’s Research Institute IACUC protocol #00096 and performed according to the institutional ethical guidelines for animal care and use. Mice were housed in the Seattle Children’s Research Institute vivarium and supplied with standard food, water and bi-weekly inspection to ensure that they were not under pain or distress throughout the duration of the experiments. Mammary fat pad injections were carried out under full anesthesia using Isoflurane gas, and mice were injected intraperitoneally with Ketoprofen 2-5mg/kg. Depth of anesthesia was verified using the toe pinch method to ensure lack of reactivity and that rodents were fully anesthetized prior to commencement of any surgical procedures. Bupivacaine 0.025% (100 uL) was administered subcutaneously to infiltrate the incisional area at the start of the surgical procedure. Mice were monitored daily for one week post surgery to ensure they were not in pain or distress. The routine method of euthanasia for mice was inhalation of carbon dioxide followed by cervical dislocation. This method of euthanasia is humane and rapid and is consistent with the recommendations of the IACUC.

### Murine tumor models

8-week-old female NOD.CB17-*Prkdc*^*scid*^/J mice (Jackson Laboratories) were used for orthotopic injections of modified 4T1 tumor cells. We had 10 mice per group for the p120-catenin WT and S/T6A injected groups and 4 mice per group in 4T1 parental and shp120 injected controls. Following anesthesia, a small incision was made to expose mammary gland number 4, and 1×10^4^ 4T1 cells per mouse were injected into the mammary fat pad. Weekly caliper measurements were used to calculate tumor volume for primary tumor growth rates. Tumor volume was estimated using Volume = (Length x Width^2^)/ 2, a common calculation to approximate tumor volume of mammary fat pad tumors, an approximation for the volume of a sphere (4/3 x *π* x r^3^). Mice were sacrificed 28 days post injection as this timepoint was shown to have established metastases based on previous experiments. At the time of sacrifice, the primary mammary tumors were formalin-fixed, paraffin-embedded, and sectioned for immunohistochemical analysis. Lungs were collected and filled with Bouin’s solution to facilitate surface tumor detection. Lung tissues were then formalin-fixed, paraffin-embedded, and sectioned for immunohistochemical analysis.

### Histology

Lung tissue and primary tumor was formalin fixed, embedded in paraffin blocks, and cut into 6 μm sections. For histology, sections were rehydrated with xylenes and a decreasing ethanol series and then stained with Mayer’s Hematoxylin (Sigma) and Eosin. Number of lung metastases were determined by counting the number of tumors in 8 sections (~ every 200 microns) per lung.

### Statistical analysis

Statistical analysis was carried using GraphPad Prism v8. Results were analyzed by one-way ANOVA followed by Tukey’s multiple comparison tests. Results from individual pair-wise comparisons are presented in the supplementary data. To compare multiple Wildtype and S/T6A p120-catenin clones, we performed two-way ANOVA analysis. P values are represented by asterisks where: * ≤0.05, ** ≤ 0.01, *** ≤ 0.001 and ****≤0.0001.

## Results

### E-cadherin activation triggers dephosphorylation of p120-catenin

**Fig 1:**
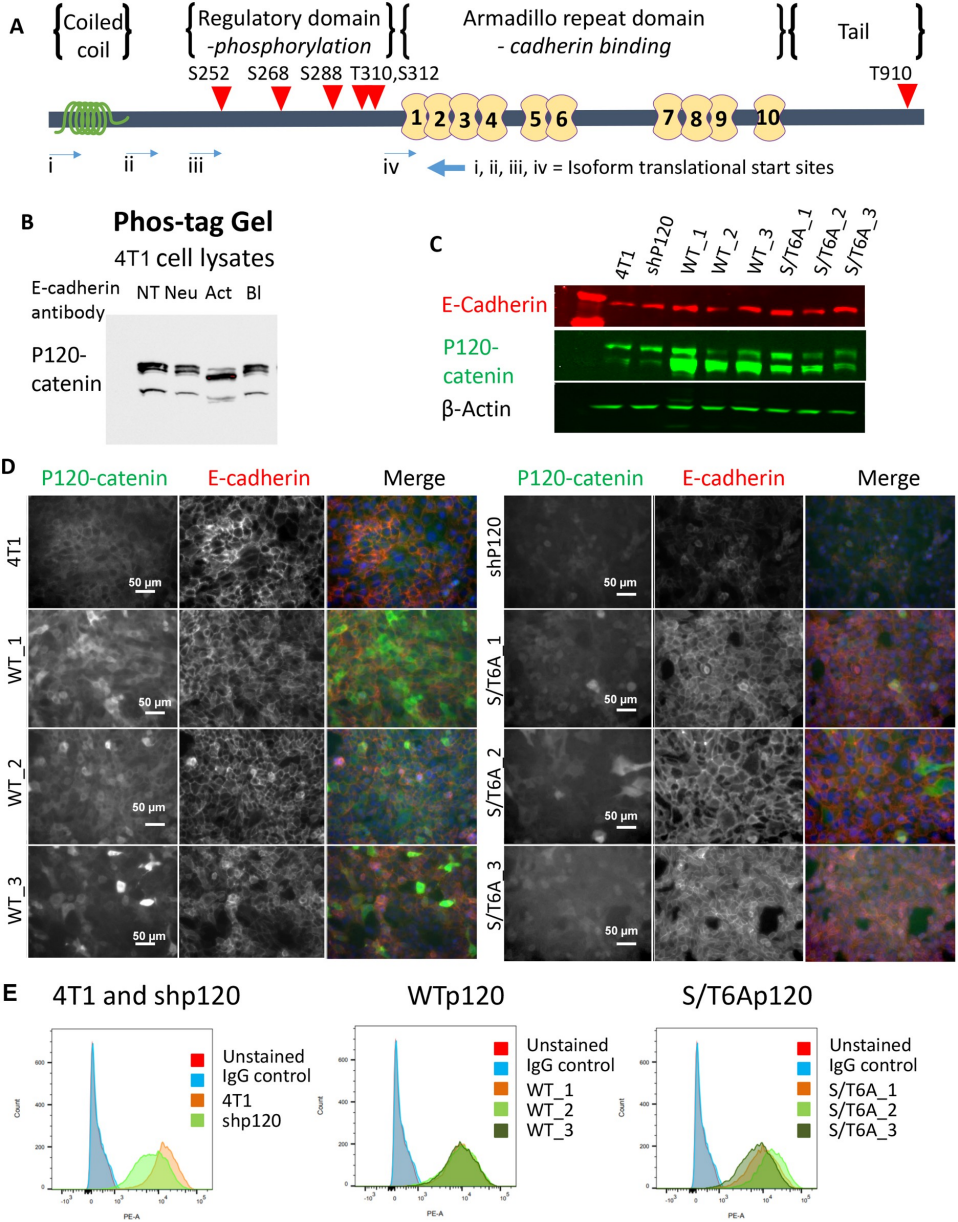
Establishment of p120-catenin phosphorylation dead mutant 4T1 cell lines. (A) Schematic showing the location of the 6 mutated phosphorylation sites in p120-catenin, indicated by red triangles. 5 of the ser/thr sites are located in the N-terminal putative regulatory domain, and 1 is in the C-terminal tail domain. (B) Decrease in p120-catenin phosphorylation observed in 4T1 cells when treated with different E-cadherin functional antibodies (3μg/ml) using a phos-tag gel. (NT: No Treatment, Neu: Neutral Antibody, Act: Activating Antibody, Bl: Blocking Antibody) (C) Expression of p120-catenin and E-cadherin protein levels by western blot in 6S/T>A phospho-mutants (S/T6A_1, S/T6A_2, S/T6A_3) or Wildtype p120-catenin isoform 3 (WT_1, WT_2, WT_3) expressing 4T1 cell lines established from single cell clones after knockdown of endogenous p120-catenin with shRNA (shP120). (D) Immunofluorescent staining of p120-catenin and E-cadherin in the 4T1 clonal cell lines established. (E) Flow cytometry staining showing rescue of surface E-cadherin levels of expression after re-expression of either WT or S/T6A p120-catenin.

The phosphorylation state of p120-catenin has been associated with the homophilic binding strength of E-cadherin at the cell surface. Previous work with Colo 205 cell activation by treatment with human E-cadherin activating antibodies, low-level trypsin, Lithium Chloride or staurosporine showed that adhesion activation was associated with a decrease in the apparent molecular weight of p120-catenin on SDS–PAGE and implicated p120-catenin dephosphorylation in E-cadherin adhesion activation [12, 19]. To determine whether p120-catenin phosphorylation is altered with E-cadherin activation in tumor cells with the ability to metastasize, we treated the murine 4T1 breast cancer cells with anti-mouse E-cadherin functional monoclonal antibodies (mAb) (described in Na et al. [28]). We decided to use the 4T1 murine breast cancer cell line as this cell line has been well documented to undergo tumor metastases while retaining E-cadherin cell surface expression [26, 27]. Treatment with E-cadherin activating antibody resulted in a decrease in apparent molecular weight of p120-catenin using a phos-tag gel, which allows increased separation of phosphorylated proteins on SDS-PAGE (Fig 1B). The decrease in phosphorylation was specific to mAb mediated E-cadherin activation, because functionally neutral and blocking mAbs that bind to E-cadherin did not cause the downward shift in band size. Thus, E-cadherin activating mAbs specifically affect p120-catenin dephosphorylation upon direct binding to the cadherin ectodomain and therefore act like agonists for this adhesion-associated event. This leads to the questions of whether p120-catenin dephosphorylation is needed for E-cadherin mediated adhesion activation and can p120-catenin dephosphorylation enhance E-cadherin adhesion strength or is the dephosphorylation of p120-catenin a consequence of enhanced E-cadherin adhesive activity.

### Expression of p120-catenin phosphorylation dead mutant alters adhesion of 4T1 murine mammary tumor cells

In order to determine whether changes in p120-catenin phosphorylation could enhance E-cadherin surface adhesion, we established clonal stable 4T1 cell lines with p120-catenin knocked down using shRNA (shP120), and restored expression with either Wildtype (WT) p120-catenin isoform 3 or a p120-catenin dephosphorylation mutant (Fig 1). The p120-catenin phosphorylation dead mutant was designed with 6 Serine or Threonine residues (S/T6A) in the regulatory domain of p120-catenin mutated to alanine which cannot be phosphorylated, thus mimicking the dephosphorylated form of p120-catenin (Fig 1A). Knockdown of p120-catenin led to a decrease in cell surface expression of E-cadherin, while all forms of p120, including phosphorylation mutants, restored normal levels of E-cadherin expression (Fig 1D and 1E). Thus, the S/T^>A phosphorylation mutations did not alter the effect of p120-catenin on E-cadherin stability or surface expression.

**Fig 2:**
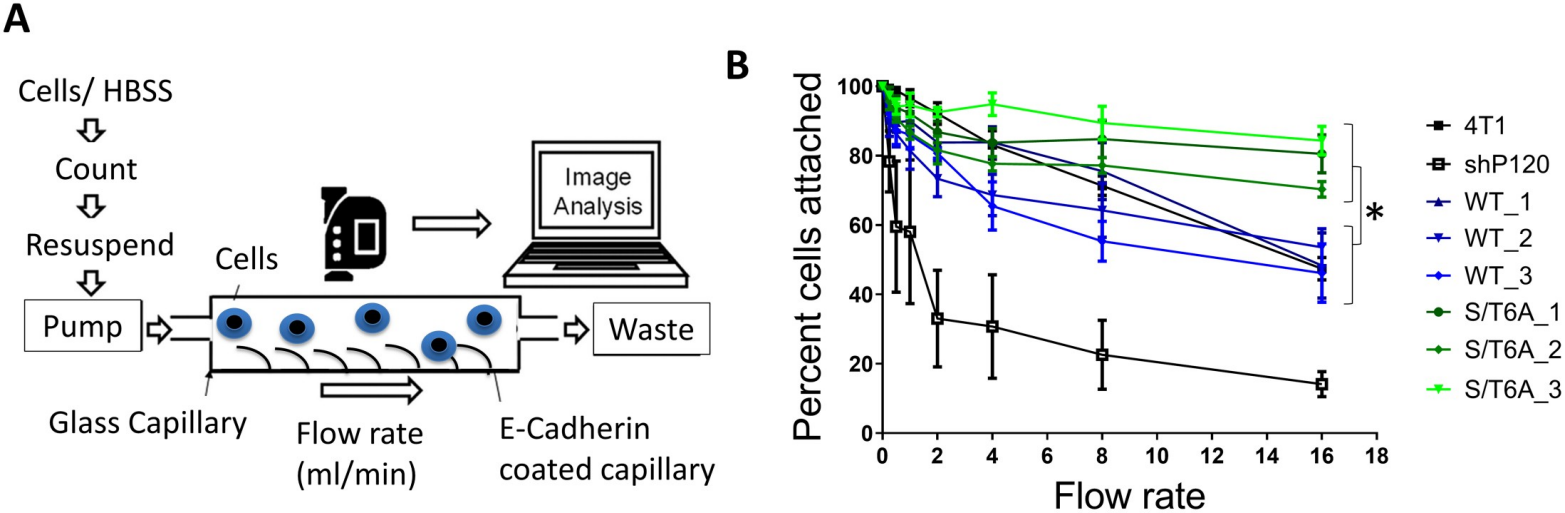
Expression of p120-catenin phosphorylation mutant alters E-cadherin mediated adhesion of 4T1 murine mammary tumor cells. (A) Schematic of the flow adhesion assay. Capillaries are coated with E-cadherin extracellular domain. Cells are allowed to adhere to the coated capillaries for 10 minutes. Hank’s Balanced Salt Solution (HBSS) is then pumped with a syringe infusion pump at different flow rates and the number of cells left attached to the capillaries are determined. (B) Graph showing the percentage of cells that remain attached to E-cadherin coated capillaries when exposed to increasing flow rates of HBSS. Cells expressing p120-catenin S/T6A mutants have increased adhesion strength as determined by their resistance to detachment under increasing flow force. Results were analyzed by two-way ANOVA followed by Tukey’s multiple comparison test. P value = 0.02.

To determine whether the change in p120-catenin phosphorylation altered the strength of adhesion in these cells, we used a flow adhesion assay and assessed the percentage of cells that remained adhered to an E-cadherin extracellular domain coated capillary when different amounts of shear force were applied by increasing flow rate using an effusion pump. E-cadherin mediated adhesion was strongly abrogated compared to parental 4T1 cells when p120-catenin was knocked down (Fig 2). This could be explained by the decrease in E-cadherin cell surface expression with loss of p120-catenin. Re-expression of WT p120-catenin restored adhesion strength similar to that of the parental 4T1 cell lines. Cells expressing the S/T6A p120-catenin mutant demonstrated increased adhesion to the E-cadherin extracellular domain coated surface while expressing equal amounts of surface E-cadherin. This demonstrated that preventing phosphorylation of p120-catenin led to increased E-cadherin mediated adhesion.

### Inability to phosphorylate p120-catenin leads to decrease in migratory and invasive properties of cells *in-vitro*

**Fig 3:**
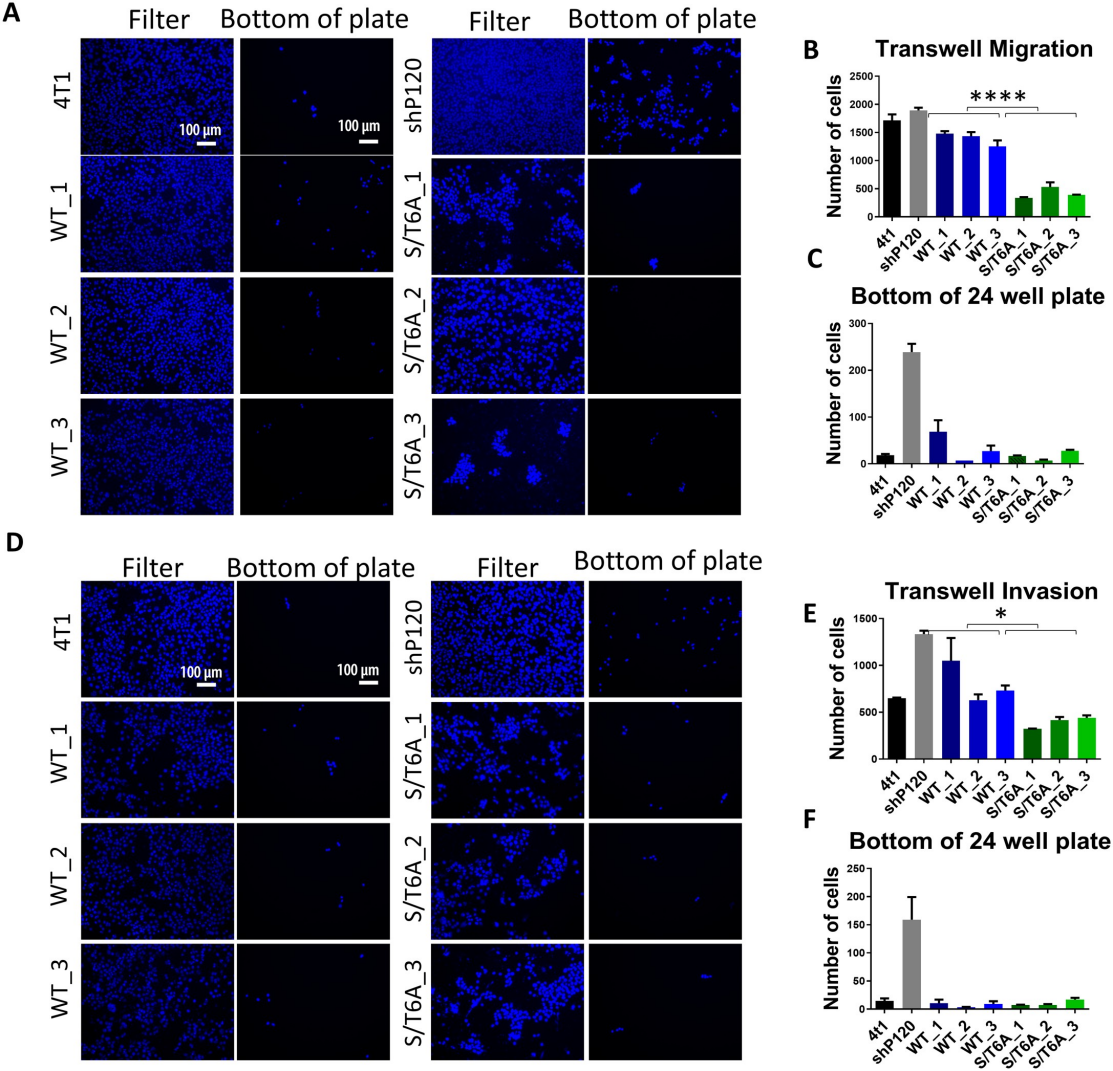
Effect of expression of p120-catenin phosphorylation dead mutant on tumor cell migration and invasion *in-vitro*. (A) Representative images of cells migrated to the bottom of the filter and bottom of the 24 well plate in a transwell migration assay. Images were taken at 10X magnification and Scalebar = 100 μm. Quantification of cells migrated to the (B) bottom of the filter and (C) bottom of the plate when stimulated with 10% FBS after 48 hours. (D) Representative images of cells invading through Basement Membrane Extract to the bottom of the filter and bottom of the 24 well plate in a transwell invasion assay. Images were taken at 10X magnification and Scalebar = 100 μm. Quantification of cells invaded to the (E) bottom of the filter and (F) bottom of the plate when stimulated with 10% FBS after 48 hours. Experiments were done in duplicate and repeated 3 times. Data was analyzed using one-way ANOVA followed by Tukey’s multiple comparison tests (S1 Table) followed by two-way ANOVA between WT and S/T6A clones. P values are represented by asterisks where: * ≤0.05 and ****≤0.0001.

After observing that expression of the p120-catenin phosphorylation dead S/T6A mutant led to an increase in cell adhesion, we were interested to understand whether these changes in cell adhesion could alter the ability of tumor cells to proliferate and affect other cancer related phenotypes *in-vitro*. *In-vitro*, re-expression of the WT or p120 S/T6A mutant did not have a significant effect on cell proliferation as determined by the MTS viability assay, BrdU incorporation and colony formation assay (S1 Fig) in the 4T1 cells. To study the effect of the phosphorylation state of p120-catenin on cell migration, control WT and p120 S/T6A mutant expressing 4T1 cells were seeded in a modified Boyden chamber. The p120 S/T6A mutant cell lines showed a 3-fold decrease in the number of cells undergoing transwell cell migration compared with the control p120-catenin WT cells after 48 hours (Fig 3A and 3B). Additionally, on looking at the bottom of the 24 well plate, while loss of p120-catenin expression led to an increase in the number of cells that fell to the bottom of the plate compared to parental 4T1 cells, re-expression of both WT or the S/T6A p120-catenin mutant reverted this phenotype (Fig 3C).

Next, the we evaluated whether the p120-catenin phosphorylation state had an effect on the ability of the tumor cell to invade through a layer of Basement Membrane Extract coated on top of the filter using the modified Boyden chamber. The p120 S/T6A expressing cells demonstrated a 2-fold decrease in invasive ability compared to those expressing WT p120-catenin (Fig 3D and 3E). Again, the number of cells that fell to the bottom of the plate was increased with knockdown of p120-catenin and restored upon expression of either p120-catenin WT or S/T6A mutant (Fig 3F).

**Fig 4:**
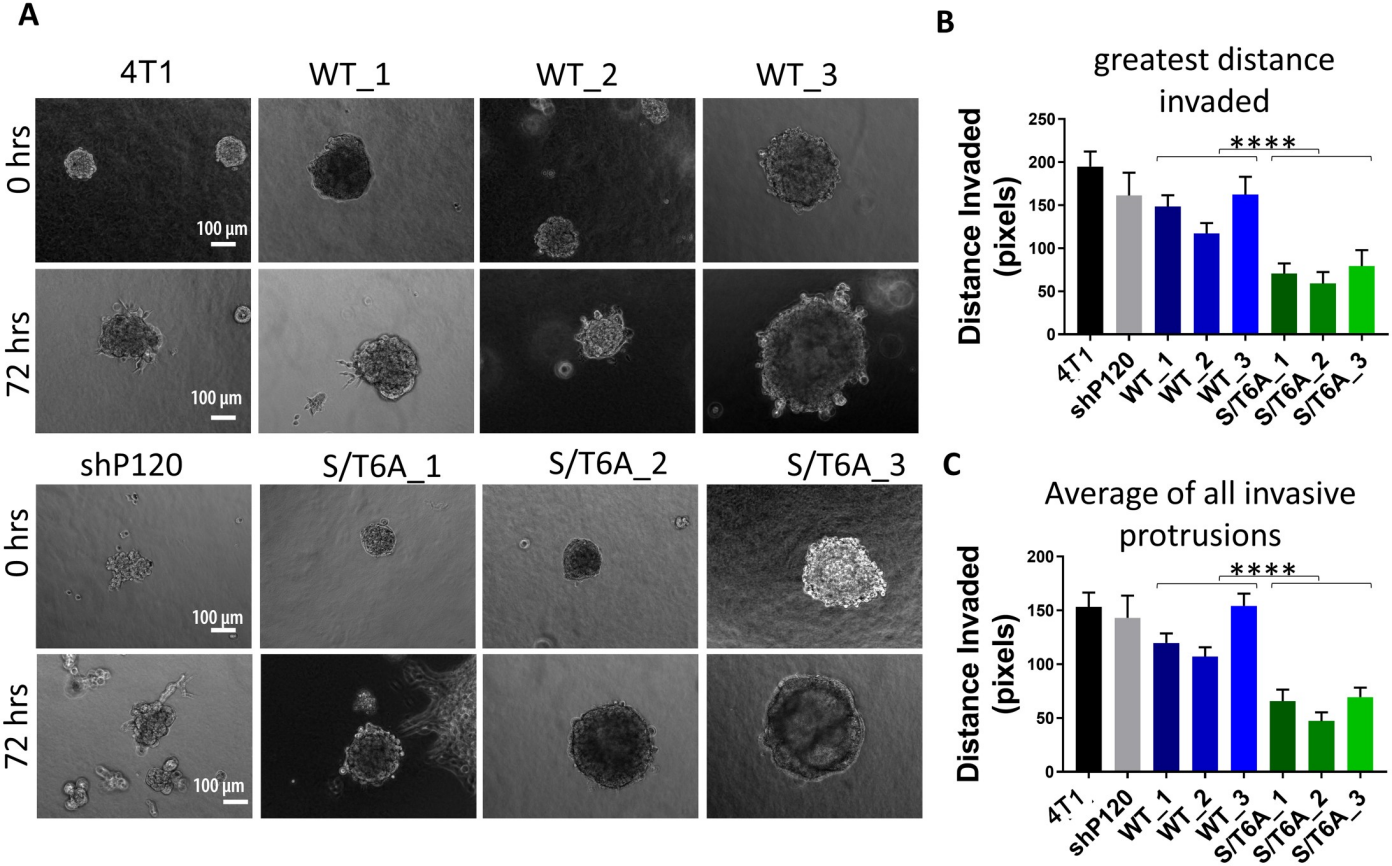
Expression of p120-catenin phosphorylation dead mutant leads to decreased tumor cell invasion in 3D collagen/matrigel matrix. (A) Representative images showing tumor cell spheroids at 0 and 72 hours post embedding in a 1:1 mixture of collagen and matrigel matrix (Not the same spheroids). Images were taken at 10X magnification and Scalebar = 100 μm. (B) Quantification of the maximum distance invaded per spheroid measured from the circumference of the spheroid in pixels through the 3D matrix (one measurement per spheroid, n = 30 per group) and (C) quantification of average distance invaded by every invasive protrusion (multiple distances per spheroid, n>30 per group). Data was analyzed using one-way ANOVA followed by Tukey’s multiple comparison tests (S2 Table) followed by two-way ANOVA between WT and S/T6A clones. P values are represented by asterisks where ****≤0.0001.

Since the effect on tumor cell invasion was less than that of the transwell migration and could be result of differences in rate of cell migration rather that an effect on the invasive ability of the cells, we further evaluated the ability of the tumor cells to invade using a 3D spheroid invasion assay that determined the ability of tumor cell spheroids to invade thorough a 1:1 mixed collagen and Matrigel extracellular matrix (Fig 4). Using this assay, we observed a decrease in the ability of tumor cells to invade as measured by distance invaded when p120-catenin is dephosphorylated compared to wildtype p120-catenin.

### Expression of p120-catenin phosphorylation dead mutant reduces response to growth factor induced cell migration

**Fig 5:**
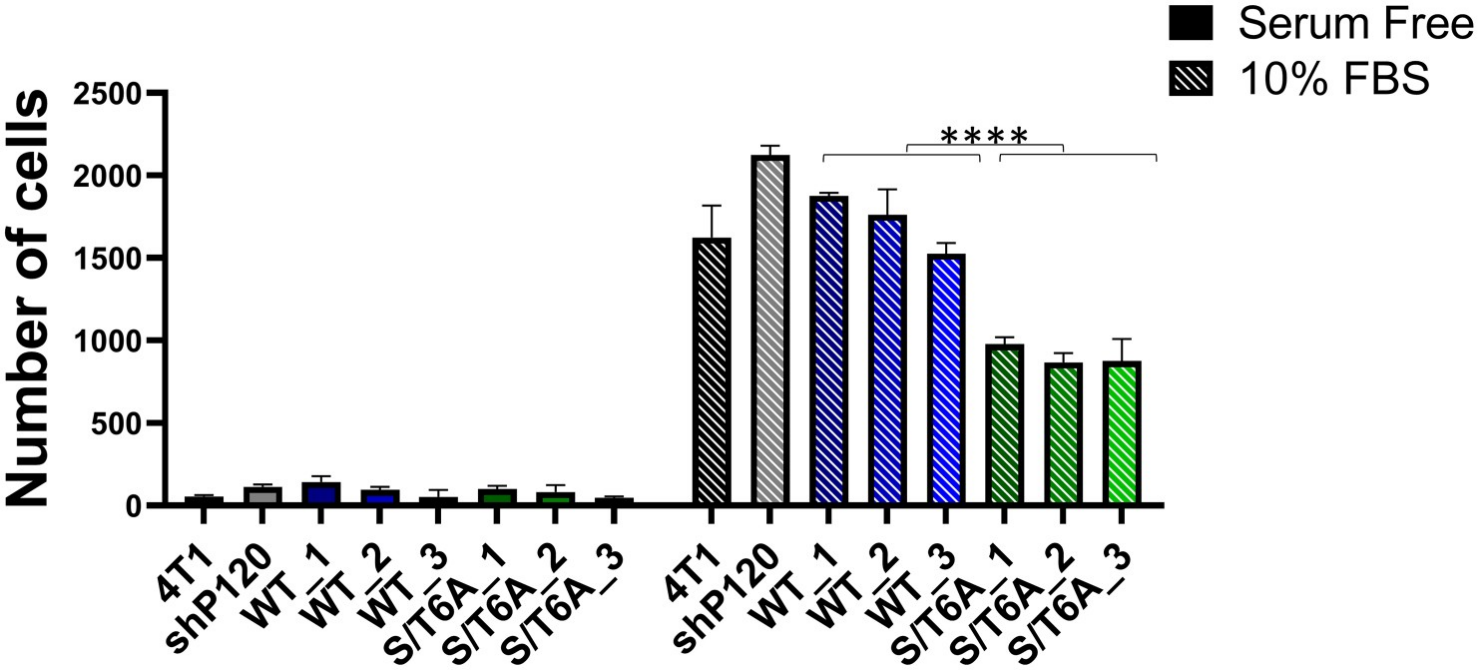
Effect of p120-catenin phosphorylation state on cell migration in response to growth factors. Quantification of the number of cells migrated to the bottom of the filter in a transwell migration assay when the lower chamber is filled with 10% FBS media versus serum free media. Data was analyzed using one-way ANOVA followed by Tukey’s multiple comparison tests (S3 Table) followed by two-way ANOVA between WT and S/T6A clones. P values are represented by asterisks where: * ≤0.05 and ****≤0.0001.

To evaluate whether this observed change in tumor cell migration and invasion was a result of the ability of the tumor cells to respond to growth factors, we assessed the difference in ability of tumor cells to migrate in serum free conditions versus when stimulated with 10% FBS (Fig 5). The results show while there is no significant difference in ability of the different cell lines to migrate under serum free conditions, we see a significant decrease in cell migration with the p120 S/T6A dephosphorylation mutant compared to cells lines expressing WT p120-catenin when stimulated with 10% FBS supplemented media. This suggests that dephosphorylation of p120-catenin leads to a decrease in the ability of tumor cells to respond to growth factor stimulation.

## Expression of p120-catenin phosphorylation dead mutant decreases the ability of tumor cells to metastasize *in-vivo*

**Fig 6:**
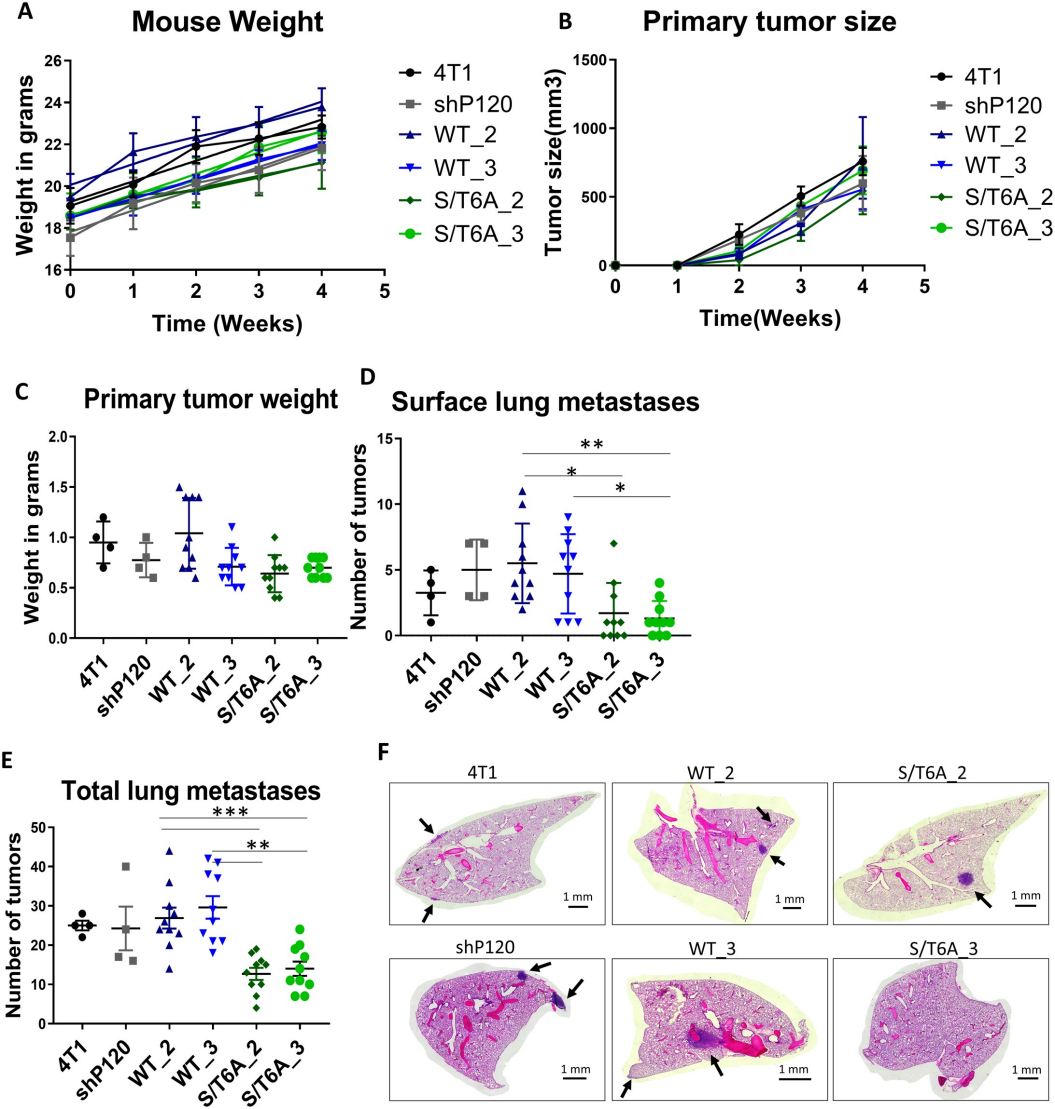
Expression of p120-catenin phosphorylation dead mutant leads to decreased tumor cell metastasis *in-vivo*. (A) Graph showing no difference in mouse weight over time post tumor cell injection. (B) Graph showing growth of primary tumor size over time measured with calipers and estimated using Volume = (Length x Width^2^)/ 2. (C) Weight in grams of primary tumor at 4 weeks post injection showed no significant difference between WT and S/T6A p120-catenin expressing tumors. Number of (D) surface lung metastases or (E) total number of tumors determined through H&E staining showing a decrease in metastatic ability of 4T1 cells expressing the S/T6A versus WT p120-catenin. (F) Representative images of lung H&E sections. Images were taken at 5X magnification and tiled together to visualize an entire lung lobe. Arrows indicate metastatic nodules. Scalebar = 1mm. Statistical analysis was carried out using GraphPad Prism software. Results were analyzed by one-way ANOVA followed by Tukey’s multiple comparison test (S4 Table). P values are represented by asterisks where: * ≤ 0.05, ** ≤ 0.01, and *** ≤ 0.001.

To test the importance of changes in p120-catenin phosphorylation on tumor progression and metastasis *in-vivo*, we used the orthotopic mammary fat pad injection model which allows us to evaluate both the primary tumor development and ability of tumor cells to metastasize (Fig 6). Using this injection model, we injected 1 × 10^4^ 4T1 cells expressing either WT or S/T6A p120-catenin directly into the mammary fat pad of NOD/SCID mice. Primary tumor growth was monitored and measured weekly using calipers. 28 days post injection, mice were sacrificed, and we harvested both the primary tumor and lungs to assess metastasis. The tumor weight and number of surface nodules on the lungs were measured at the time of sacrifice (Fig 6C and 6D). Lung tissues were processed for histology and 8 sections, 200 microns apart, per lung were stained by H&E to record the incidence of metastasis (Fig 6F). Overall, there was a 50% reduction in tumor metastases in mice injected with p120 S/T6A expressing cells compared to control WT p120-catenin cell lines, even though there was no discernable effect on primary tumor size (Fig 6E and 6A). These results show that dephosphorylation of p120-catenin leads to a decrease in the ability of tumor cells to metastasize with no difference in primary tumor growth and development.

## Discussion

E-cadherin and the catenin complex are integral to the maintenance and function of the normal epithelial cells and are frequently dysregulated in cancer [31, 32]. E-cadherin homophilic binding at the cell surface is essential for cell-cell adhesion [29, 33]. p120-catenin has been proposed to act as a tumor suppressor by virtue of its ability to stabilize E-cadherin at the cell surface and prevent its endocytosis [13]. Loss of expression or mutations that alter its adhesive function have been associated with EMT and the metastatic progression of cancer [1, 31]. Although transcriptional regulation is most often responsible for E-cadherin expression loss, there is evidence that cadherins can be regulated rapidly and post translationally [32]. Previous work from our lab have shown that both xenopus C-cadherin and human E-cadherin adhesive activity can be regulated independently of the surface expression levels and that changes in p120-catenin phosphorylation state could be one of such mechanisms [19, 34].

p120-catenin has been shown to be phosphorylated in several types of cancer [18], but the effect on this phosphorylation state on E-cadherin mediated cell adhesion has not been previously evaluated. Previous studies have implied that when E-cadherin adhesive activity was enhanced, either through use of E-cadherin activating antibodies or use of actin stabilizers, bound p120-catenin was in the de-phosphorylated state [11, 12]. In this paper we show that the phosphorylation state of p120-catenin is important in mediating changes in E-cadherin adhesion strength in metastatic tumor cells. We show that by altering the phosphorylation of p120-catenin by mutating six Serine and Threonine residues to Alanine that mimic the phosphorylation dead or dephosphorylated form of p120-catenin, we can enhance E-cadherin mediated cell adhesion in 4T1 metastatic tumor cells. We further show that when p120-catenin is in the dephosphorylated state, there is a decrease in the metastatic cancer phenotype of tumor cells both *in-vitro* and *in-vivo*. Thus, identifying key kinases that control the phosphorylation state of p120-catenin can play an important role in the ability to treat and control cancer from metastasizing.

There are several conditions that can alter p120-catenin phosphorylation. Several growth factors and kinases including Src, EGF and PKC have been shown to phosphorylate p120-catenin at multiple sites [15, 17, 23, 35]. Levels of T916 phosphorylation are elevated in renal cell carcinoma, even though they showed that T916 phosphorylation was not essential in the transforming ability of the tumor cells [18]. This suggests that changes in p120-catenin phosphorylation at a single residue might not be sufficient for cancer transformation of cells on its own but a result of collective effects at multiple phosphorylation sites. Indeed, we did not see significant alterations in E-cadherin adhesive activity when single Serine/Threonine residues of p120-catenin were mutated to Alanine compared to the six residue S/T6A mutant [11]. Activation of Wnt signaling can induce phosphorylation of p120-catenin at S268 and S269 [36], which was claimed to dissociate p120-catenin from E-cadherin and subsequently led to activation of downstream Wnt signaling events. S288 phosphorylation levels are elevated in lung cancer cell lines and tumor tissues as well [37].

The changes in p120-catenin phosphorylation that alter E-cadherin mediated cell adhesion could explain instances of metastasis in tumor cells that still retain a high level of E-cadherin expression. The loss, mutation or decrease in E-cadherin levels was previously thought to be essential for the metastatic spreading of tumor cells [2, 3]. However recent studies have shown that this is not necessarily the case and maintenance of E-cadherin is important in the collective migration of tumor cells and facilitate survival of tumor cells in the blood [6–8]. Rapid and small changes in E-cadherin binding strength, as a result of changes in p120-catenin phosphorylation/ dephosphorylation could be integral to this process.

In summary, our findings show that in addition to stabilizing E-cadherin at the cell surface, the p120-catenin phosphorylation state can control cell adhesive strength. The dephosphorylation of p120-catenin, when bound to E-cadherin can decrease the metastatic progression of cancer by virtue of its ability to increase cell adhesion by enhancing the E-cadherin adhesive activity state at the cell surface. This result has important implications for understanding the roles of p120-catenin phosphorylation and cadherins in tissue morphogenesis and cancer progression. It will be important to understand how p120-catenin phosphorylation is regulated and controlled and how subtle alterations in E-cadherin adhesive function can impact cancer progression and metastasis.

## Conclusion

Cancer Metastasis is responsible for majority of the morbidity and mortality associated with cancer. While each type of cancer is different, the decrease or loss of E-cadherin expression is commonly observed in cancers that have metastasized. This study provides an understanding of the mechanisms that control E-cadherin homophilic binding at the cell surface through changes in p120-catenin phosphorylation. The results from this study show that p120-catenin dephosphorylation enhances the binding strength of E-cadherin, increasing cell adhesion and decreasing cancer metastasis. Since E-cadherin regulation in Cancer is not limited to any one cancer type, approaches to alter E-cadherin activation could be more broadly applicable to all types of cancer.

## Supporting information

S1 Fig Effect of p120 de-phosphorylation on tumor cells on cell growth *in vitro*. Knockdown of p120 catenin with shRNA or expression of the 6S/T>A dephosphorylation mutant in 4T1 cells did not cause significant difference in cell proliferation as determined by (A) MTT assay or (B) BrdU proliferation assay. (C) Representative images of colony formation assay showing no significant differences in the ability of tumor cells to form colonies in soft agar.
(TIF)

S1 Table Pairwise tukey test results after one-way ANOVA analysis of tumor cell migration and invasion *in-vitro*. Table showing P values after one-way ANOVA analysis and Tukey’s multiple comparison tests for tumor cell migration and invasion (through a layer of Matrigel) to the bottom of the filter and the bottom of the 24 well plate.
(DOCX)

S2 Table Pairwise tukey test results after one-way ANOVA analysis of 3D tumor cell invasion *in-vitro*. Table showing P values after one-way ANOVA analysis and Tukey’s multiple comparison tests for distance invaded per spheroid or of all invasive protrusions measured in pixels.
(DOCX)

S3 Table Pairwise tukey test results after one-way ANOVA analysis of tumor cell migration in response to 10% FBS. Table showing P values after one-way ANOVA analysis and Tukey’s multiple comparison tests for tumor cell migration to the bottom of the filter when the lower chamber is filled with serum free media versus 10% FBS.
(DOCX)

S4 Table Pairwise tukey test results after one-way ANOVA analysis of primary tumor growth and metastases *in-vivo*. Table showing P values after one-way ANOVA analysis and Tukey’s multiple comparison tests for primary tumor weight, surface lung metastases and total lung metastases after orthotopic mammary fat pad injection of 4T1 tumor cells.
(DOCX)

S1 File Uncropped gels from Fig 1. (PDF)

## References

[1] Mendonsa AM, Na TY, Gumbiner BM. E-cadherin in contact inhibition and cancer. Oncogene. 2018;37(35):4769–80. 10.1038/s41388-018-0304-2 29780167 PMC6119098

[2] Onder TT, Gupta PB, Mani SA, Yang J, Lander ES, Weinberg RA. Loss of E-cadherin promotes metastasis via multiple downstream transcriptional pathways. Cancer research. 2008;68(10):3645–54. 10.1158/0008-5472.CAN-07-2938 18483246

[3] Hanahan D, Weinberg RA. Hallmarks of cancer: the next generation. Cell. 2011;144(5):646–74. 10.1016/j.cell.2011.02.013 21376230

[4] Lamouille S, Xu J, Derynck R. Molecular mechanisms of epithelial-mesenchymal transition. Nature reviews Molecular cell biology. 2014;15(3):178–96. 10.1038/nrm3758 24556840 PMC4240281

[5] Berx G, van Roy F. Involvement of members of the cadherin superfamily in cancer. Cold Spring Harbor perspectives in biology. 2009;1(6):a003129 10.1101/cshperspect.a003129 20457567 PMC2882122

[6] Cheung KJ, Gabrielson E, Werb Z, Ewald AJ. Collective invasion in breast cancer requires a conserved basal epithelial program. Cell. 2013;155(7):1639–51. 10.1016/j.cell.2013.11.029 24332913 PMC3941206

[7] Cheung KJ, Ewald AJ. A collective route to metastasis: Seeding by tumor cell clusters. Science (New York, NY). 2016;352(6282):167–9. 10.1126/science.aaf6546 PMC8183671 27124449

[8] Padmanaban V, Krol I, Suhail Y, Szczerba BM, Aceto N, Bader JS, et al E-cadherin is required for metastasis in multiple models of breast cancer. Nature. 2019;573(7774):439–44. 10.1038/s41586-019-1526-3 31485072 PMC7365572

[9] Corso G, Marrelli D, Roviello F. Familial gastric cancer and germline mutations of E-cadherin. Annali italiani di chirurgia. 2012;83(3):177–82. 22595728

[10] Hansford S, Kaurah P, Li-Chang H, Woo M, Senz J, Pinheiro H, et al Hereditary Diffuse Gastric Cancer Syndrome: CDH1 Mutations and Beyond. JAMA oncology. 2015;1(1):23–32. 10.1001/jamaoncol.2014.168 26182300

[11] Maiden SL, Petrova YI, Gumbiner BM. Microtubules Inhibit E-Cadherin Adhesive Activity by Maintaining Phosphorylated p120-Catenin in a Colon Carcinoma Cell Model. PLoS One. 2016;11(2):e0148574 10.1371/journal.pone.0148574 26845024 PMC4742228

[12] Petrova YI, Spano MM, Gumbiner BM. Conformational epitopes at cadherin calcium-binding sites and p120-catenin phosphorylation regulate cell adhesion. Mol Biol Cell. 2012;23(11):2092–108. 10.1091/mbc.E11-12-1060 22513089 PMC3364174

[13] Davis MA, Ireton RC, Reynolds AB. A core function for p120-catenin in cadherin turnover. The Journal of cell biology. 2003;163(3):525–34. 10.1083/jcb.200307111 14610055 PMC2173649

[14] Fukumoto Y, Shintani Y, Reynolds AB, Johnson KR, Wheelock MJ. The regulatory or phosphorylation domain of p120 catenin controls E-cadherin dynamics at the plasma membrane. Experimental cell research. 2008;314(1):52–67. 10.1016/j.yexcr.2007.07.024 17719574 PMC2211447

[15] Reynolds AB, Herbert L, Cleveland JL, Berg ST, Gaut JR. p120, a novel substrate of protein tyrosine kinase receptors and of p60v-src, is related to cadherin-binding factors beta-catenin, plakoglobin and armadillo. Oncogene. 1992;7(12):2439–45. 1334250

[16] Mariner DJ, Anastasiadis P, Keilhack H, Bohmer FD, Wang J, Reynolds AB. Identification of Src phosphorylation sites in the catenin p120ctn. The Journal of biological chemistry. 2001;276(30):28006–13. 10.1074/jbc.M102443200 11382764

[17] Xia X, Mariner DJ, Reynolds AB. Adhesion-associated and PKC-modulated changes in serine/threonine phosphorylation of p120-catenin. Biochemistry. 2003;42(30):9195–204. 10.1021/bi034597h 12885254

[18] Kourtidis A, Yanagisawa M, Huveldt D, Copland JA, Anastasiadis PZ. Pro-Tumorigenic Phosphorylation of p120 Catenin in Renal and Breast Cancer. PLoS One. 2015;10(6):e0129964 10.1371/journal.pone.0129964 26067913 PMC4466266

[19] Shashikanth N, Petrova YI, Park S, Chekan J, Maiden S, Spano M, et al Allosteric Regulation of E-Cadherin Adhesion. The Journal of biological chemistry. 2015;290(35):21749–61. 10.1074/jbc.M115.657098 26175155 PMC4571897

[20] Schackmann RC, Klarenbeek S, Vlug EJ, Stelloo S, van Amersfoort M, Tenhagen M, et al Loss of p120-catenin induces metastatic progression of breast cancer by inducing anoikis resistance and augmenting growth factor receptor signaling. Cancer research. 2013;73(15):4937–49. 10.1158/0008-5472.CAN-13-0180 23733751

[21] Short SP, Kondo J, Smalley-Freed WG, Takeda H, Dohn MR, Powell AE, et al p120-Catenin is an obligate haploinsufficient tumor suppressor in intestinal neoplasia. The Journal of clinical investigation. 2017;127(12):4462–76. 10.1172/JCI77217 29130932 PMC5707165

[22] Smalley-Freed WG, Efimov A, Short SP, Jia P, Zhao Z, Washington MK, et al Adenoma formation following limited ablation of p120-catenin in the mouse intestine. PLoS One. 2011;6(5):e19880 10.1371/journal.pone.0019880 21611205 PMC3096651

[23] Dohn MR, Brown MV, Reynolds AB. An essential role for p120-catenin in Src- and Rac1-mediated anchorage-independent cell growth. The Journal of cell biology. 2009;184(3):437–50. 10.1083/jcb.200807096 19188496 PMC2646551

[24] Huveldt D, Lewis-Tuffin LJ, Carlson BL, Schroeder MA, Rodriguez F, Giannini C, et al Targeting Src family kinases inhibits bevacizumab-induced glioma cell invasion. PLoS One. 2013;8(2):e56505 10.1371/journal.pone.0056505 23457577 PMC3572988

[25] Ma LW, Zhou ZT, He QB, Jiang WW. Phosphorylated p120-catenin expression has predictive value for oral cancer progression. Journal of clinical pathology. 2012;65(4):315–9. 10.1136/jclinpath-2011-200516 22259181

[26] Petrova YI, Schecterson L, Gumbiner BM. Roles for E-cadherin cell surface regulation in cancer. Mol Biol Cell. 2016;27(21):3233–44. 10.1091/mbc.E16-01-0058 27582386 PMC5170857

[27] Lou Y, Preobrazhenska O, auf dem Keller U, Sutcliffe M, Barclay L, McDonald PC, et al Epithelial-mesenchymal transition (EMT) is not sufficient for spontaneous murine breast cancer metastasis. Developmental dynamics: an official publication of the American Association of Anatomists. 2008;237(10):2755–68. 18773493 10.1002/dvdy.21658

[28] Tae-Young Na LS, 1 Alisha M. Mendonsa,1 and Barry M. Gumbiner1,2,3. E-cadherin activating monoclonal antibodies inhibit breast cancer metastasis in mice at multiple steps in the metastatic cascade. PNAS. 2019.

[29] Chappuis-Flament S, Wong E, Hicks LD, Kay CM, Gumbiner BM. Multiple cadherin extracellular repeats mediate homophilic binding and adhesion. The Journal of cell biology. 2001;154(1):231–43. 10.1083/jcb.200103143 11449003 PMC2196848

[30] Berens EB, Holy JM, Riegel AT, Wellstein A. A Cancer Cell Spheroid Assay to Assess Invasion in a 3D Setting. J Vis Exp. 2015(105). 10.3791/53409 PMC4692745 26649463

[31] Jeanes A, Gottardi CJ, Yap AS. Cadherins and cancer: how does cadherin dysfunction promote tumor progression? Oncogene. 2008;27(55):6920–9. 10.1038/onc.2008.343 19029934 PMC2745643

[32] Gumbiner BM. Regulation of cadherin-mediated adhesion in morphogenesis. Nature reviews Molecular cell biology. 2005;6(8):622–34. 10.1038/nrm1699 16025097

[33] Takeichi M. Morphogenetic roles of classic cadherins. Current opinion in cell biology. 1995;7(5):619–27. 10.1016/0955-0674(95)80102-2 8573335

[34] Zhong Y, Brieher WM, Gumbiner BM. Analysis of C-cadherin regulation during tissue morphogenesis with an activating antibody. The Journal of cell biology. 1999;144(2):351–9. 10.1083/jcb.144.2.351 9922460 PMC2132887

[35] Mariner DJ, Davis MA, Reynolds AB. EGFR signaling to p120-catenin through phosphorylation at Y228. Journal of cell science. 2004;117(Pt 8):1339–50. 10.1242/jcs.01001 14996911

[36] Del Valle-Perez B, Casagolda D, Lugilde E, Valls G, Codina M, Dave N, et al Wnt controls the transcriptional activity of Kaiso through CK1epsilon-dependent phosphorylation of p120-catenin. Journal of cell science. 2011;124(Pt 13):2298–309. 10.1242/jcs.082693 21670201

[37] Zhang PX, Wang Y, Liu Y, Jiang GY, Li QC, Wang EH. p120-catenin isoform 3 regulates subcellular localization of Kaiso and promotes invasion in lung cancer cells via a phosphorylation-dependent mechanism. International journal of oncology. 2011;38(6):1625–35. 10.3892/ijo.2011.995 21468542

