# Supplementary figures and images for: p120-catenin phosphorylation status alters E-cadherin mediated cell adhesion and ability of tumor cells to metastasize
Source: PLoS One. 2020 Jun 26;15(6):e0235337. doi: 10.1371/journal.pone.0235337 (PMC7319294; doi:10.1371/journal.pone.0235337)

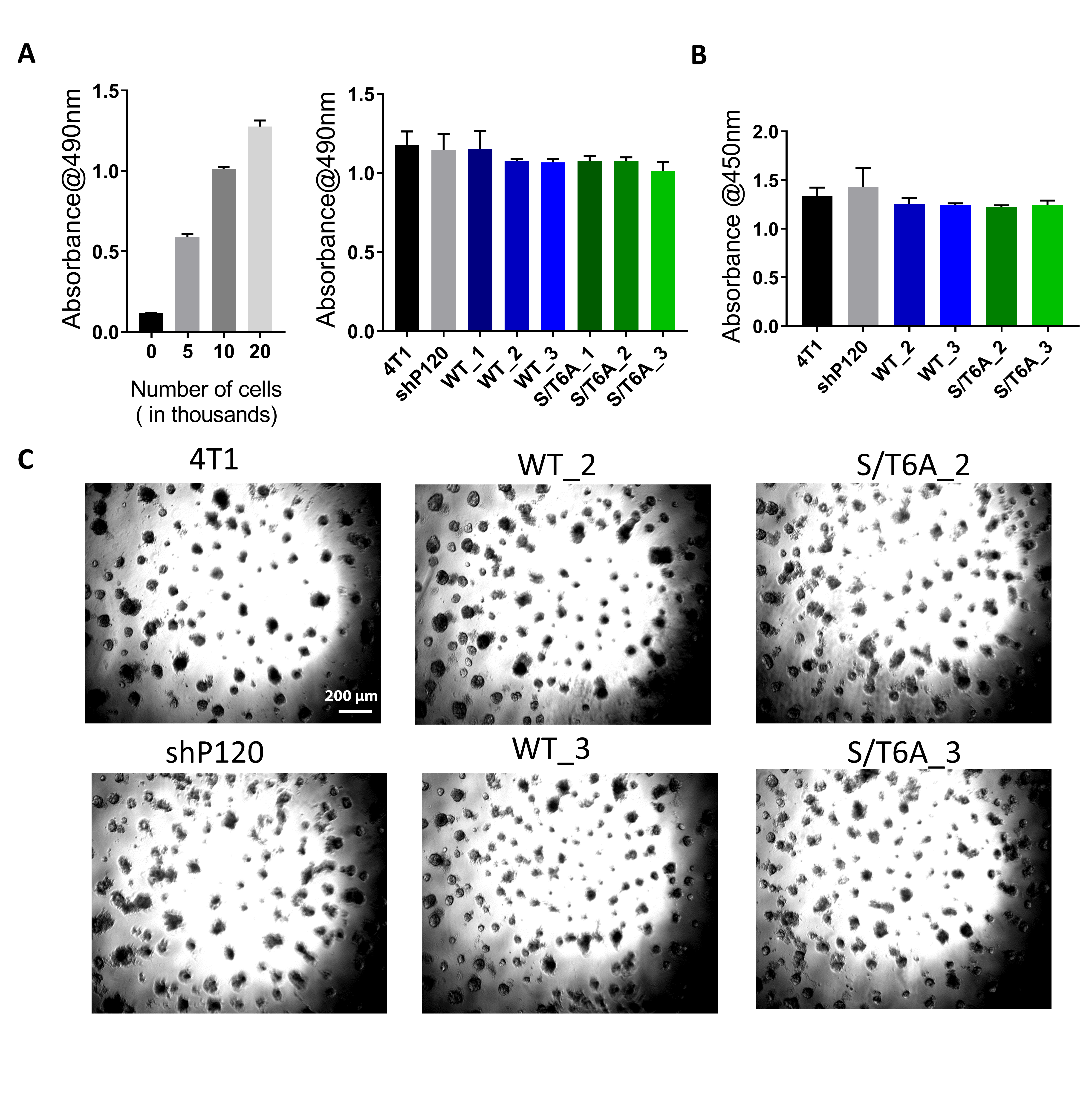

Supplement: S1 Fig — Knockdown of p120 catenin with shRNA or expression of the 6S/T>A dephosphorylation mutant in 4T1 cells did not cause significant difference in cell proliferation as determined by (A) MTT assay or (B) BrdU proliferation assay. (C) Representative images of colony formation assay showing no significant differences in the ability of tumor cells to form colonies in soft agar. (TIF) [file pone.0235337.s001.tif]

**Fig 1A**

**Phos-tag Gel**

4t1 cell lysates

E-cadherin  
antibody

NT Neu Act Bl

P120-  
catenin

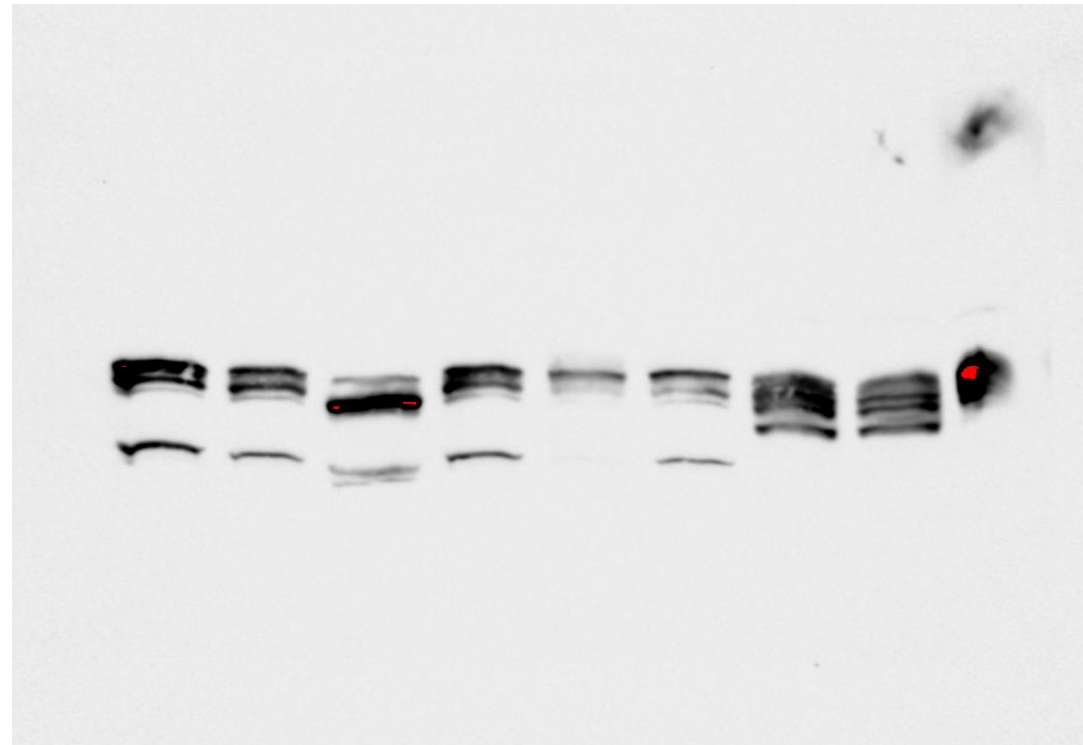

**Fig 1B**

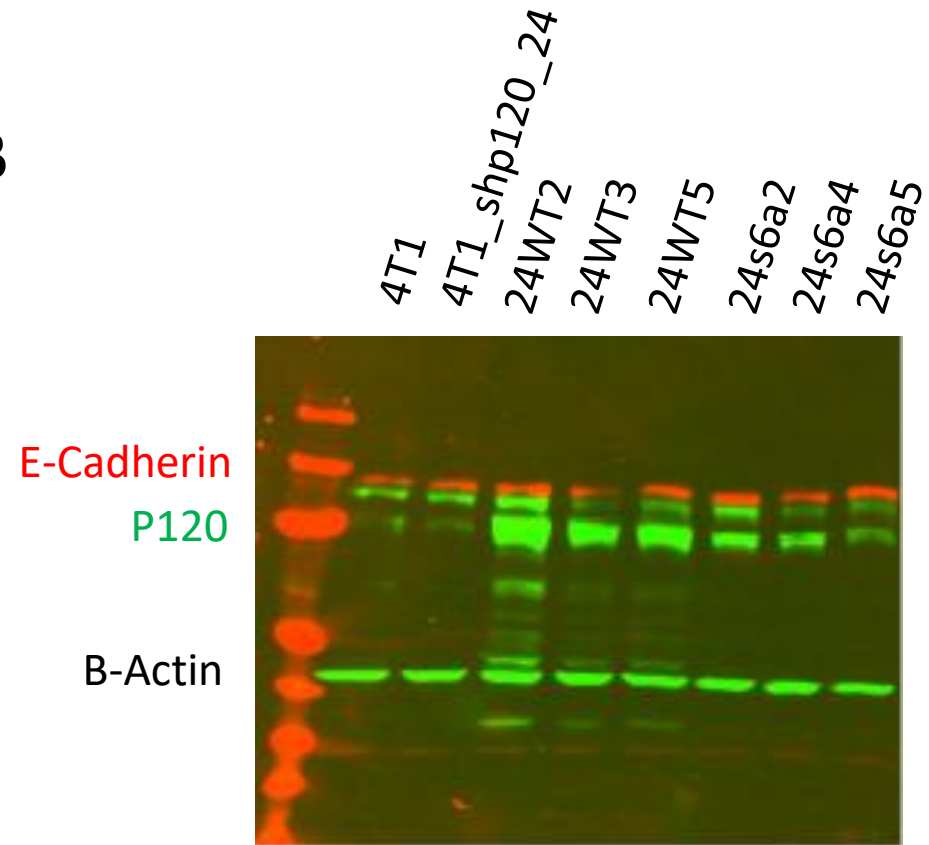

Supplement: S1 File — (PDF) [file pone.0235337.s006.pdf]
