# Supplementary material for: p120-catenin phosphorylation status alters E-cadherin mediated cell adhesion and ability of tumor cells to metastasize
Source: PLoS One. 2020 Jun 26;15(6):e0235337. doi: 10.1371/journal.pone.0235337 (PMC7319294; doi:10.1371/journal.pone.0235337)
Supplement: S1 Table — Table showing P values after one-way ANOVA analysis and Tukey’s multiple comparison tests for tumor cell migration and invasion (through a layer of Matrigel) to the bottom of the filter and the bottom of the 24 well plate. (DOCX) [file pone.0235337.s002.docx]

**S1 Table.** **Pairwise Tukey test results after one-way ANOVA analysis of tumor cell migration and invasion *in-vitro*.**

| Tukey's multiple comparisons test | Adjusted P Value | | | |
| --- | --- | --- | --- | --- |
|  | Migration | | Invasion | |
|  | Filter | 24 well plate | Filter | 24 well plate |
| 4t1 vs. shP120 | 0.6479 | <0.0001 | 0.0109 | 0.0017 |
| 4t1 vs. WT_1 | 0.3632 | 0.1596 | 0.1604 | >0.9999 |
| 4t1 vs. WT_2 | 0.2106 | 0.9944 | >0.9999 | 0.999 |
| 4t1 vs. WT_3 | 0.0224 | 0.9991 | 0.9973 | >0.9999 |
| 4t1 vs. S/T6A_1 | <0.0001 | >0.9999 | 0.3228 | >0.9999 |
| 4t1 vs. S/T6A_2 | <0.0001 | 0.9944 | 0.6541 | >0.9999 |
| 4t1 vs. S/T6A_3 | <0.0001 | 0.9987 | 0.7468 | >0.9999 |
| shP120 vs. WT_1 | 0.0395 | <0.0001 | 0.4496 | 0.0014 |
| shP120 vs. WT_2 | 0.0223 | <0.0001 | 0.0091 | 0.001 |
| shP120 vs. WT_3 | 0.003 | <0.0001 | 0.0227 | 0.0013 |
| shP120 vs. S/T6A_1 | <0.0001 | <0.0001 | 0.0009 | 0.0012 |
| shP120 vs. S/T6A_2 | <0.0001 | <0.0001 | 0.0017 | 0.0012 |
| shP120 vs. S/T6A_3 | <0.0001 | <0.0001 | 0.002 | 0.0019 |
| WT_1 vs. WT_2 | 0.9996 | 0.0652 | 0.1325 | >0.9999 |
| WT_1 vs. WT_3 | 0.4191 | 0.3012 | 0.3408 | >0.9999 |
| WT_1 vs. S/T6A_1 | <0.0001 | 0.1422 | 0.0077 | >0.9999 |
| WT_1 vs. S/T6A_2 | 0.0002 | 0.0652 | 0.0174 | >0.9999 |
| WT_1 vs. S/T6A_3 | <0.0001 | 0.3121 | 0.0216 | >0.9999 |
| WT_2 vs. WT_3 | 0.6495 | 0.9051 | 0.9903 | >0.9999 |
| WT_2 vs. S/T6A_1 | <0.0001 | 0.9976 | 0.3828 | >0.9999 |
| WT_2 vs. S/T6A_2 | 0.0003 | >0.9999 | 0.7327 | >0.9999 |
| WT_2 vs. S/T6A_3 | <0.0001 | 0.8947 | 0.8191 | 0.9964 |
| WT_3 vs. S/T6A_1 | 0.0002 | 0.9976 | 0.1511 | >0.9999 |
| WT_3 vs. S/T6A_2 | 0.0013 | 0.9051 | 0.3544 | >0.9999 |
| WT_3 vs. S/T6A_3 | 0.0004 | >0.9999 | 0.4309 | 0.9999 |
| S/T6A_1 vs. S/T6A_2 | 0.5604 | 0.9976 | 0.9943 | >0.9999 |
| S/T6A_1 vs. S/T6A_3 | 0.9989 | 0.9967 | 0.9799 | 0.9996 |
| S/T6A_2 vs. S/T6A_3 | 0.8332 | 0.8947 | >0.9999 | 0.9996 |
