# Supplementary material for: p120-catenin phosphorylation status alters E-cadherin mediated cell adhesion and ability of tumor cells to metastasize
Source: PLoS One. 2020 Jun 26;15(6):e0235337. doi: 10.1371/journal.pone.0235337 (PMC7319294; doi:10.1371/journal.pone.0235337)
Supplement: S2 Table — Table showing P values after one-way ANOVA analysis and Tukey’s multiple comparison tests for distance invaded per spheroid or of all invasive protrusions measured in pixels. (DOCX) [file pone.0235337.s003.docx]

**S2 Table.** **Pairwise Tukey test results after one-way ANOVA analysis of 3D tumor cell invasion *in-vitro*.**

| Tukey's multiple comparisons test | Adjusted P Value | |
| --- | --- | --- |
|  | Maximum distance Migrated | Average Distance Migrated |
| 4t1 vs. shP120 | 0.8658 | 0.9995 |
| 4t1 vs. WT_1 | 0.4574 | 0.2233 |
| 4t1 vs. WT_2 | 0.0062 | 0.0228 |
| 4t1 vs. WT_3 | 0.8853 | >0.9999 |
| 4t1 vs. S/T6A_1 | <0.0001 | <0.0001 |
| 4t1 vs. S/T6A_2 | <0.0001 | <0.0001 |
| 4t1 vs. S/T6A_3 | <0.0001 | <0.0001 |
| shP120 vs. WT_1 | 0.9996 | 0.9343 |
| shP120 vs. WT_2 | 0.5879 | 0.6069 |
| shP120 vs. WT_3 | >0.9999 | 0.9994 |
| shP120 vs. S/T6A_1 | 0.0224 | 0.007 |
| shP120 vs. S/T6A_2 | 0.0032 | 0.0002 |
| shP120 vs. S/T6A_3 | 0.0364 | 0.0101 |
| WT_1 vs. WT_2 | 0.8446 | 0.9856 |
| WT_1 vs. WT_3 | 0.9994 | 0.3068 |
| WT_1 vs. S/T6A_1 | 0.0501 | 0.0153 |
| WT_1 vs. S/T6A_2 | 0.0073 | 0.0001 |
| WT_1 vs. S/T6A_3 | 0.081 | 0.0222 |
| WT_2 vs. WT_3 | 0.5558 | 0.0465 |
| WT_2 vs. S/T6A_1 | 0.4529 | 0.1514 |
| WT_2 vs. S/T6A_2 | 0.1276 | 0.0039 |
| WT_2 vs. S/T6A_3 | 0.6276 | 0.209 |
| WT_3 vs. S/T6A_1 | 0.0198 | <0.0001 |
| WT_3 vs. S/T6A_2 | 0.0028 | <0.0001 |
| WT_3 vs. S/T6A_3 | 0.0321 | <0.0001 |
| S/T6A_1 vs. S/T6A_2 | 0.9997 | 0.9569 |
| S/T6A_1 vs. S/T6A_3 | >0.9999 | >0.9999 |
| S/T6A_2 vs. S/T6A_3 | 0.9869 | 0.8771 |
