# Supplementary material for: p120-catenin phosphorylation status alters E-cadherin mediated cell adhesion and ability of tumor cells to metastasize
Source: PLoS One. 2020 Jun 26;15(6):e0235337. doi: 10.1371/journal.pone.0235337 (PMC7319294; doi:10.1371/journal.pone.0235337)
Supplement: S3 Table — Table showing P values after one-way ANOVA analysis and Tukey’s multiple comparison tests for tumor cell migration to the bottom of the filter when the lower chamber is filled with serum free media versus 10% FBS. (DOCX) [file pone.0235337.s004.docx]

**S3 Table.** **Pairwise Tukey test results after one-way ANOVA analysis of tumor cell migration in response to 10% FBS.**

| Tukey's multiple comparisons test | Serum Free | 10% FBS |
| --- | --- | --- |
| 4T1 vs. shP120 | 0.9839 | <0.0001 |
| 4T1 vs. WT_1 | 0.8641 | 0.0087 |
| 4T1 vs. WT_2 | 0.9984 | 0.3974 |
| 4T1 vs. WT_3 | >0.9999 | 0.7781 |
| 4T1 vs. S/T6A_1 | 0.9957 | <0.0001 |
| 4T1 vs. S/T6A_2 | 0.9998 | <0.0001 |
| 4T1 vs. S/T6A_3 | >0.9999 | <0.0001 |
| shP120 vs. WT_1 | 0.9997 | 0.01 |
| shP120 vs. WT_2 | >0.9999 | <0.0001 |
| shP120 vs. WT_3 | 0.9793 | <0.0001 |
| shP120 vs. S/T6A_1 | >0.9999 | <0.0001 |
| shP120 vs. S/T6A_2 | 0.9997 | <0.0001 |
| shP120 vs. S/T6A_3 | 0.9664 | <0.0001 |
| WT_1 vs. WT_2 | 0.9942 | 0.6386 |
| WT_1 vs. WT_3 | 0.8459 | 0.0001 |
| WT_1 vs. S/T6A_1 | 0.9978 | <0.0001 |
| WT_1 vs. S/T6A_2 | 0.9797 | <0.0001 |
| WT_1 vs. S/T6A_3 | 0.8032 | <0.0001 |
| WT_2 vs. WT_3 | 0.9977 | 0.0158 |
| WT_2 vs. S/T6A_1 | >0.9999 | <0.0001 |
| WT_2 vs. S/T6A_2 | >0.9999 | <0.0001 |
| WT_2 vs. S/T6A_3 | 0.9949 | <0.0001 |
| WT_3 vs. S/T6A_1 | 0.994 | <0.0001 |
| WT_3 vs. S/T6A_2 | 0.9997 | <0.0001 |
| WT_3 vs. S/T6A_3 | >0.9999 | <0.0001 |
| S/T6A_1 vs. S/T6A_2 | >0.9999 | 0.6714 |
| S/T6A_1 vs. S/T6A_3 | 0.9886 | 0.739 |
| S/T6A_2 vs. S/T6A_3 | 0.9991 | >0.9999 |
