# Supplementary material for: p120-catenin phosphorylation status alters E-cadherin mediated cell adhesion and ability of tumor cells to metastasize
Source: PLoS One. 2020 Jun 26;15(6):e0235337. doi: 10.1371/journal.pone.0235337 (PMC7319294; doi:10.1371/journal.pone.0235337)
Supplement: S4 Table — Table showing P values after one-way ANOVA analysis and Tukey’s multiple comparison tests for primary tumor weight, surface lung metastases and total lung metastases after orthotopic mammary fat pad injection of 4T1 tumor cells. (DOCX) [file pone.0235337.s005.docx]

**S4 Table.** **Pairwise Tukey test results after one-way ANOVA analysis of primary tumor growth and metastases *in-vivo*.**

| Tukey's multiple  comparisons test | Adjusted P Value | | |
| --- | --- | --- | --- |
|  | Primary tumor weight | Surface lung metastases | Total number of metastases |
| 4T1 vs. shP120 | 0.8661 | 0.9129 | >0.9999 |
| 4T1 vs. WT_2 | 0.9816 | 0.6361 | 0.9979 |
| 4T1 vs. WT_3 | 0.4448 | 0.9161 | 0.8962 |
| 4T1 vs. S/T6A_2 | 0.1822 | 0.8918 | 0.0737 |
| 4T1 vs. S/T6A_3 | 0.3992 | 0.7605 | 0.1407 |
| shP120 vs. WT_2 | 0.3351 | 0.9993 | 0.9899 |
| shP120 vs. WT_3 | 0.9958 | >0.9999 | 0.8213 |
| shP120 vs. S/T6A_2 | 0.9009 | 0.229 | 0.108 |
| shP120 vs. S/T6A_3 | 0.9919 | 0.1342 | 0.1974 |
| WT_2 vs. WT_3 | 0.0689 | 0.9773 | 0.9624 |
| WT_2 vs. S/T6A_2 | 0.0525 | 0.0149 | 0.0013 |
| WT_2 vs. S/T6A_3 | 0.0644 | 0.0054 | 0.0042 |
| WT_3 vs. S/T6A_2 | 0.9791 | 0.0903 | <0.0001 |
| WT_3 vs. S/T6A_3 | >0.9999 | 0.0383 | 0.0003 |
| S/T6A_2 vs. S/T6A_3 | 0.9895 | 0.9991 | 0.9987 |
